# Characterization of *Fusarium acuminatum*: A Potential Enniatins Producer in Tunisian Wheat

**DOI:** 10.3390/jof8050458

**Published:** 2022-04-28

**Authors:** Yasmine Chakroun, Souheib Oueslati, Laetitia Pinson-Gadais, Manef Abderrabba, Jean-Michel Savoie

**Affiliations:** 1 INRAE, UR1264 Mycology and Food Safety, MycSA, F-33882 Villenave d’Ornon, France; yasminechakroun@gmail.com (Y.C.); laetitia.pinson-gadais@inrae.fr (L.P.-G.); 2 IPEST—Laboratory Molecules Material and Applications, LMMA, University of Carthage, Tunis 2070, Tunisia; souheibo@yahoo.fr (S.O.); manef.abderrabba@ipest.rnu.tn (M.A.)

**Keywords:** mycotoxins, essential oils, molecular taxonomy, fungistatic activity

## Abstract

*Fusarium* Head Blight (FHB), caused by multiple species of *Fusarium* in small grain cereals, is a significant and long-standing problem anywhere in the world. Knowing regional *Fusarium* spp. present on non-symptomatic grains and their potential for mycotoxin production is of concern for identifying novel actions for FHB and mycotoxin management, such as treatments with essential oils. Analyzing the mycotoxin content of grains from non-symptomatic ears of different wheat varieties cultivated in Tunisia, we isolated *Fusaria* specimens identified as *F. culmorum* and *F. acuminatum* using analysis of the partial DNA sequence of the β-tubulin gene and ITS region. Two isolates of the latter species, uncommon in cereal grains in this region until now, were shown to be effective producers of enniatins in vitro, with 1390 and 3089 µg g^−1^ mycelial biomass (dry) in 11-day-old cultures. The susceptibility of an isolate of *F. acuminatum* to the fungistatic and antimycotoxin effects of eight essential oils was measured. Essential oils from *Ammoides pusilla* and *Thymus capitatus* used at 0.1 µL mL^−1^ in an agar culture medium, affected the mycelial growth by 55% and 79%, respectively and reduced the accumulation of enniatins per unit of mycelial colony by 26% and 52%, respectively. Finally, *F. acuminatum* was shown to be a contaminant of wheat grains in Tunisia and it may contribute to the contamination in enniatins. Two essential oils of Tunisian plants could be used for developing a biofungicide limiting both its mycelial growth and its accumulation of mycotoxins in grains.

## 1. Introduction

Cereal-based food crops are an important part of the culinary heritage of multiple cultures all over the world. In the Northern African region, food intakes are based mainly on cereals such as wheat, barley, maize, and sorghum consumed as raw grains, flours, or processed products. Furthermore, cereals are susceptible to fungal diseases such as Fusarium Head Blight (FHB) caused by *Fusarium* species. The disease reduces the yield of harvested cereals and impacts the quality of grains, which become starchy and inadequate for industrial processing.

The different *Fusarium* species causing this disease are mainly *F. graminearum*, *F. culmorum*, *F. pseudograminearum*, *F. avenaceum*, *F. equiseti*, and *F. poe*. These *Fusarium* species are able to produce mycotoxins defined as toxic secondary metabolites of low molecular weight, are thermally stable and have a high bioaccumulation ability in crops from sowing to harvesting in the field, but also at storage and during food processing [1]. Mycotoxins at high concentrations are potentially harmful to humans’ and animals’ health. The most known *Fusarium* mycotoxins in cereals are Type B Trichothecene (TCTB) including deoxynivalenol (DON) and its acetylated derivatives, Type A Trichothecenes, Fumonisins, and Zearalenone (ZEA). They have a wide range of harmful effects such as carcinogenicity, teratogenicity, and mutagenicity [2]. A less known *Fusarium* mycotoxins group, called “emergent mycotoxins”, is receiving increasing interest from researchers. Enniatins (ENNs), Beauvericin, and Moniliformin mainly make up this group [3]. Although no acute toxicity was reported from the exposure to these mycotoxins, in vitro studies demonstrated their cytotoxicity on different cell lines, their ability to impair cell cycle distribution, to cause mitochondrial dysfunction, and to induce apoptosis. Moreover, their presence in large amounts and their natural co-presence with other mycotoxins may induce synergistic effects [4]. Among the 29 identified ENNs, four analogs are the most detected in cereal grains, enniatin A (ENA), enniatin A1 (ENA1), enniatin B (ENB), and enniatin B1 (ENB1) [4]. Most of the *Fusarium* strains that have been reported to produce these ENNs in cereals are part of the *F. sambucinum* species complex (i.e., *F. venenatum* and *F. poae*) as well as the *F. tricinctum* species complex composed by *F. tricinctum*, *F. avenaceum*, *F. torulosum*, and *F. acuminatum* [5].

Several *Fusarium* species have been found in cereals cultivated or consumed in Tunisia. Studying FHB symptomatic heads at the filling stage from 11 wheat fields in Northern Tunisia during the 2004 and 2007 crop seasons, these were found as the main *Fusarium* species: *F. culmorum*, *F. avenaceum*, and *F. equiseti* [6]. In another study during the 2004 crop season on infested durum wheat spikes, it was reported that the most common species isolated were *Microdochium nivale* var *nivale* (63.5%), *F. culmorum* (26%), *F. pseudograminearum* (9%), and *F. avenaceum* (1.5%) [7]. When determining the mycoflora of wheat and barley grain samples consumed in Tunisia in 2011, Jedidi et al. [8] isolated *Fusarium* species from 18% of wheat and 50% of barley samples, with a mean percentage of infected grains per sample of 1.17% and 1.1% in wheat and barley grains, respectively. The main *Fusarium* species they isolated was *F. equiseti*. To the best of our knowledge, no report has been conducted about the isolation of *F. acuminatum* from cereals produced in Tunisia. It is obvious that the prevalent *Fusarium* species found in Tunisian wheat grains are producers of TCTB or ENNs. However, in a multi-mycotoxin analysis in 34 wheat and 5 barley samples of asymptomatic grains from Tunisian markets in 2009, Oueslati et al. [9] did not detect TCTB. In another study focused on the detection of emerging mycotoxins, it was shown that 96% of the analyzed wheat and barley samples were contaminated with different ENNs [10].

Key global actions for mycotoxin management in wheat and other small grains have recently been reviewed [11]. The use of fungicides is one of them. The efficiency depends on the active molecule, the application timing, method and rate, the cereal variety, and the populations and species of *Fusarium* present [11]. However, to limit the accumulation of pesticide residues in the environment and in food, and the dependency on the companies producing them, alternatives to synthetic fungicides using local resources are actively searched for in various countries. The use of non-toxic and eco-friendly plant essential oils (EOs) is one alternative. Some EOs have been shown to be effective against *Fusarium* species and their production of mycotoxins [12,13,14,15]. Finding alternatives to synthetic fungicides for controlling local populations of *Fusarium* spp. causing mycotoxin contamination of wheat and small grain cereals is of concern in Tunisia and other Mediterranean countries, as a contribution to the improvement in food safety.

The present work aimed at improving the knowledge on the contamination of non-symptomatic Tunisian wheat grains by *Fusarium* species and mycotoxins (both regulated and emerging groups), and to shed light on *F. acuminatum*, a rarely found species that had never been associated with ENNs production in Tunisian cereals before. A strain isolated from the studied Tunisian wheat grains was characterized as a potential source of ENNs contamination. Its susceptibility to EOs from eight Tunisian plants was assessed.

## 2. Materials and Methods

### 2.1. Wheat Samples

Twenty-three samples of wheat grain from various varieties were randomly collected at harvest time at the locality of Béjà (North West Tunisia), representing six durum wheat varieties (Karim, Khiar, Maâli, Nasr, Om Rabii, Razzek) and four bread wheat varieties (Byrsa, Hidra, Salambôo, Utique) on two years, 2017 and 2018, and three additional bread wheat varieties (Vaga, Tahent, Badii) in 2017. All the samples were stored in hermetic polyethylene bags at room temperature until mycotoxins analysis and isolation of *Fusarium* species.

### 2.2. Mycotoxin Extraction from Wheat Grains

Mycotoxin extraction from grains was adapted from a previously published method [16]. The homogenized grains were dried at 50 °C for 48 h, then ground into a fine powder (particles size <100 µm). Five g of powder were extracted with 20 mL acetonitrile/water (84:16, *v/v*) rotating end-over-end at 50 rpm for 60 min, followed by centrifugation (4 °C, 10 min at 3000× *g*) and the supernatant was collected. The fraction was either directly evaporated to dryness under a gentle nitrogen stream for further analysis of ENNs or purified through a trichothecene P^®^ column (R-Biopharm, Darmstadt, Germany) and 4 mL of the eluate was collected before drying by evaporation for further analysis of TCTBs and ZEA.

### 2.3. Mycotoxin Analysis—TCTB and ZEA

The dried extracts were resuspended in methanol/water (1:1, *v/v*) and filtered through a 0.2 µm filter (Phenomenex, Le Peck, France) for the quantification of ZEA and TCTBs, including DON and its 15 and 3 acetylated derivatives (3-ADON and 15-ADON), Nivalenone (NIV), and its derivative, Fusarenon X (FUS-X), using an Agilent Technologies 1100 series HPLC chain, equipped with an Agilent photodiode array detector (DAD) and the ChemStation chromatography manager software (Agilent, Waldbronn, Germany). The injection volume was 10 µL. Separation was achieved on a Kinetex XB-C18 100 Å column (150 × 4.6 mm^2^, 2.6 μm) (Phenomenex, Le Peck, France) maintained at 45 °C. The mobile phase consisted of acidified water with ortho-phosphoric acid, pH 2.6 (solvent A), and acetonitrile (solvent B), and the following elution gradient was used: starting at 93% A and 7% B for 1 min, 93% to 70% A and 7 to 30% B in 10 min, 70 to 10% A and 30 to 90% B in 10 min, 10 to 93% A and 90 to 7% B in 2 min, and 5 min post-run equilibration with the initial conditions. The flow rate was kept at 1 mL min^−1^ for a total run time of 27 min. UV-VIS spectra were recorded from 220 to 550 nm and peak areas were measured at 230 nm. Quantification was performed using external calibration ranging from 6.25 to 100 µg mL^−1^ prepared with standard solutions (Romer Labs, Tulln, Austria). The limit of quantification was 0.1 µg g^−1^ grain for each of the mycotoxins.

### 2.4. Mycotoxin Analysis—ENNs

For the quantification of ENNs, the dried extracts were resuspended in acetonitrile/water (85:15, *v/v*), vortexed, filtered through 0.45 µm filters, and 10 µL was injected in an Agilent Technologies 1100 series HPLC chain, equipped with an Agilent photodiode array detector (DAD) (Agilent, Waldbronn, Germany) for separation on a Zorbax Eclipse XDB-C8 (4.6 × 15 mm^2^, 5 µm) maintained at 30 °C. The mobile phase consisted of MilliQ water (solvent A) and acetonitrile (solvent B). The flow was kept at 1 mL min^−1^ for a total run time of 26 min. The elution gradient was: 30% A and 70% B for 5 min, 30 to 10% A and 70 to 90% B in 10 min, 10% A and 90% B for 5 min, 10 to 30% A and 90 to 70 % B in 1 min, 30% A and 70% B for 5 min. Absorbance spectra were recorded from 190 to 450 nm and peak areas were measured at 205 nm. Quantification was performed using external calibration ranging from 1 to 100 µg mL^−1^ prepared with standard solutions of enniatin A (ENA), enniatin A1 (ENA1), enniatin B (ENB), enniatin B1 (ENB1) (Sigma-Aldrich, Saint-Quentin-Fallavier France). The limit of detection was 0.01 µg g^−1^ of grain.

Alternatively, to check the presence/absence of the targeted mycotoxins measured with the HPLC/DAD method, five samples collected in 2018 were randomly selected for extractions and LC-MS/MS analysis performed as in Ducos et al. [16] using a QTrap 2000 system (AB Sciex, Courtaboeuf, France) equipped with an Electro Spray Ionization (ESI) source and an 1100 Series HPLC system (Agilent, Les Ulis, France), as an alternative to the HPLC-DAD analysis presented above. Calibration in the matrix was performed using pure standards of ENA, ENA1, ENB, ENB1 (Sigma-Aldrich, France) diluted at 10 to 250 ng mL^−1^. Retention times were 6.35, 6.28, 6.11, 6.20 min for ENA, ENA1, ENB, ENB1, respectively, and their precursor ions were 699.49, 685.48, 5657.44, and 671.46 m/z, respectively. The limit of detection was 0.8 ng g^−1^ of grain

### 2.5. Fusarium Selection, Isolation and Microscopic Characterization

The sample’s contamination with *Fusarium* species was after surface sterilization. A total of 100 grains alternatively selected from each sample were soaked in 10% sodium hypochlorite for 5 min and rinsed 3 times with sterile deionized water before being placed on potato dextrose agar (PDA) medium. After incubation for 3 to 5 days at 25 °C, the mycelial growth was examined and plugs from the different mycelial colonies were transferred to new PDA plates. Monosporic cultures were obtained by suspension dilutions followed by isolation under a stereomicroscope of one germinated spore per isolate. The monosporic cultures were morphologically identified according to Leslie and Summerell keys [17]. The macroscopic examination of the cultures of the fungal species was carried out on PDA and on FDM-agar medium the composition of which was: 12.5 g of glucose, 4.25 g of NaNO_3_, 5 g of NaCl, 2.5 g of MgSO_4_·7H_2_O, 1.36 g of KH_2_PO_4_, 0.01 g of FeSO_4_·7H_2_O, 0.0029 g of ZnSO_4_·7H_2_O, 15 g of agar, 1 L of water [18]. The characteristics of micro- and macroconidia (appearance of the conidiophore, form, and size) were observed under an optical microscope (magnifications: 100×, 2000×).

### 2.6. Molecular Characterization and Taxonomy

Four monosporic isolates were grown on PDA for 7 days for collecting mycelium, which was freeze-dried and ground to a fine powder with the Tissue Lyser System^®^ (Qiagen, Les Ulis, France). For the fungal DNA extraction, the NucleoMag 96 Plant Macherey-Nagel Kit (Macherey-Nagel Gmbh & Co. KG, Dueren, Germany) was used according to the supplier’s instructions. Briefly, 500 µL of MC1 lyse buffer and 10 µL of RNAse were added to 50 mg of ground mycelium, then vigorously vortexed. The samples were incubated for 30 min at 56 °C, centrifuged at 16,000× *g* for 20 min. Meanwhile, a plate was prepared within the first line, 100 µL of the binding buffer MC2 and 15 µL of the NucleoMAg c-Beads were added into each well. In the wells of the second line, 200 µL of washing buffer MC3 was added. In the wells of third line, 200 µL of washing buffer MC4 was added. In the wells of the fourth line, 200 µL of an 80% solution of EtOH was added, 200 µL of washing buffer MC5 in the fifth line and, finally, 80 µL of MC6 elution buffer in the last line. In total, 100 µL of each sample solution was added in the wells of the first line of the plate and the latter was positioned into the MagMax^TM^ Express Robot (Applied Biosystems, Espoo, Finland). The extracted DNA was finally eluted in the last line of the plate. The quality and quantity of the extracted DNA were assessed both by 1% agarose gel electrophoresis and by UV spectrophotometry using a nanodrop^®^ ND 1000 spectrophotometer (Labtech International LTD©, Uckfield, UK).

Further identification of *Fusarium* species was performed by PCR amplification targeting the nucleotide sequences of the transcribed spacer sites (ITS) and the β-tubulin (*β-tub*) gene. The amplification of these loci was carried out with the primers listed in Table 1. The PCR mix contained 15.25 µL of PCR-grade water, 5 µL of KAPA HIFI Buffer (5×); 0.75 µL of 10 mM KAPA dNTP Mix, 0.75 µL of each primer at 10 µM, 2 µL of the template DNA at 20 ng µL^−1^, and 0.5 µL of 1 U µL^−1^ of KAPA HIFI DNA polymerase for a total reactional volume of 25 µL. The PCR cycle was conducted as follows: 3 min at 95 °C for the initial denaturation, followed by 25 cycles of 20 s at 98 °C for the denaturation phase, 45 s at the primer’s annealing temperature (58 °C), 60 s at 72 °C for the elongation and, finally, 2 min at 72 °C for the final elongation.

The PCR product obtained was sequenced by the company GENEWIZ (Leipzig, Germany). Nucleotide sequences were aligned using the Clustal W algorithm in BioEdit 7.0 software. Sequence similarity searches were performed with BLAST (basic local alignment search tool) against NCBI public database (www.ncbi.nlm.nih.gov/genbank, 15 March 2022). Further, dendrograms were constructed using the MEGA-X program (MEGA-X: Molecular Evolutionary Genetics Analysis version 10.0.5 (Tamura, Stecher, and Kumar, 1993–2020) and original sequences of target species and nucleotide sequences (target and other species of the genus *Fusarium*) from the NCBI database were used: original nucleotide sequences of DNA sites (Beta Tubulin)—*F. acuminatum* (MH341248), *F. avenaceum* (EU357849), *F. tricinctum* (MK936369), *F. culmorum* (KP765705), and *F. graminearum* (MN416026). In addition, *Aspergillus carbonarius* (HE577803) was used as an outer group. The sequences of the four isolates from this study have the following numbers in GeneBank: 3T = ON128289, 4T = ON128290, 32T = ON128291, 33T = ON128292. The data are presented as a consensus phylogenetic tree using the unweighted pair group (UPGMA) method. Branches corresponding to partitions reproduced in less than 50% bootstrap replicates are collapsed. The percentage of replicate trees in which the associated taxa clustered together in the bootstrap test (100 replicates) are shown next to the branches. The evolutionary distances were computed using the Maximum Composite Likelihood method and are in the units of the number of base substitutions per site. This analysis involved 10 nucleotide sequences. Codon positions included were 1st + 2nd + 3rd + Noncoding. All ambiguous positions were removed for each sequence pair (pairwise deletion option).

### 2.7. In Vitro Growth and Production of Enniatins in Agar Media

Plugs were taken from each previously prepared monosporal culture and incubated in liquid CMC medium (15 g carboxymethyl cellulose, 1 g yeast extract, 0.5 g MgSO_4_·7H_2_O, 1 g NH_4_NO_3_, 1 g KH_2_PO_4_, 1 L distilled water) for 48 h to induce sporulation. Ten µL of 10^6^ mL^−1^ spore suspension was inoculated on 10 mL of PDA or FDM-agar, which was previously described as an ENNs-inducing medium [18]. The FDM-agar medium composition was: 12.5 g of glucose, 4.25 g of NaNO_3_, 5 g of NaCl, 2.5 g of MgSO_4_·7H_2_O, 1.36 g of KH_2_PO_4_, 0.01 g of FeSO_4_·7H_2_O, 0.0029 g of ZnSO_4_·7H_2_O, 15 g of agar, 1 L of distilled water. The cultures were incubated for 11 days and the growth was measured as the surface covered by the mycelium using ImageJ software. The mycotoxins were extracted on the last day of culture. The plate content (agar medium and mycelium) was sliced and placed into 35 mL ethyl acetate (VWR, Fontenay-Sous-Bois, France) for 15 min at room temperature rotating end-over-end at 250 rpm and filtrated on No 4 Whatman filter paper. Five mL were evaporated to dryness at 45 °C under a gentle nitrogen flow. The dried samples were resuspended with 200 µL of methanol/water (1:1, *v/v*) and filtered on 0.2 µm filter before ENNs’ analysis using the HPLC-DAD method as in Section 2.4.

### 2.8. In Vitro Growth and Production of Enniatins in Liquid FDM

Alternatively, for assessing the production of ENNs, liquid cultures in FDM were also used as in Gautier et al. [4], with incubation for 11 d at 25 °C. After centrifugation at 5000× *g* and filtration, 4 mL was sampled and 8 mL of ethyl acetate was added in 15 mL PTFE tubes. The mixture was vortexed for 2 min and agitated for 15 min at 250 rpm as above. Then, 5 mL of the supernatant was evaporated to dryness at 45 °C under a nitrogen flow. The dried samples were dissolved with 200 µL of methanol/water (1:1, *v/v*) and filtered on 0.2 µm filter before ENNs’ analysis using the HPLC-DAD method as in Section 2.4. The mycelium collected after centrifugation was freeze-dried and weighted.

### 2.9. Assessment of the Fusarium Susceptibility to Essential Oils

The effects of EOs from eight Tunisian plants (Ammoides pusilla, Artemisia absintum, Carum carvi, Mentha spicata, Myrtus communis, Origanum vulgare, Schinus terbentofonius, Thymus capitatus) on the mycelial growth and production of ENNs by one of the isolates, F. acuminatum 33T, were measured in FDM-agar as described by Chakroun et al. [15] and in Section 2.7. Briefly, the EOs were diluted at 0.1 µL mL^−1^ in FDM-agar medium poured into 90 mm Petri dishes. After central inoculation with a spore suspension and incubation for 10 d at 25 °C, the surface colonized by the mycelium was measured and the contents of the plates were collected for ENNs’ extraction and analysis using the HPLC-DAD method.

### 2.10. Statistical Analyses

Mycelial growth and ENNs concentrations were analysed by ANOVA and Duncan’s multiple range tests using SAS Software (Statistical Analysis System, version 9, Cary, NC, USA). Differences were considered at a significance level of 95% (*p* < 0.05).

## 3. Results

### 3.1. Mycotoxin Contamination of the Wheat Grain Samples

None of the following mycotoxins were detected in the 23 analyzed grain samples, ZEA, NIV, FUS-X, 3-ADON, and 15-ADON, while DON was detected in only one sample, the Khiar variety collected in 2018, at a concentration of 320 µg Kg^−1^. This variety was shown to be highly susceptible to FHB [6].

No ENA1 nor ENB were detected, but all the samples were positive for ENA and ENB1 analyzed with HPLC-DAD. The ENA level measured was 10-fold higher than the ENB1 level (Table 2). No significant differences between the collection years and the type of wheat were observed for both ENA and ENB1 (5%, *t*-test).

None of the samples were positive for ENA or ENB1 when analysed with HPLC-MS/MS.

### 3.2. Fusarium Strains Isolated from Grains of Tunisian Wheat Varieties

**Figure 1:**
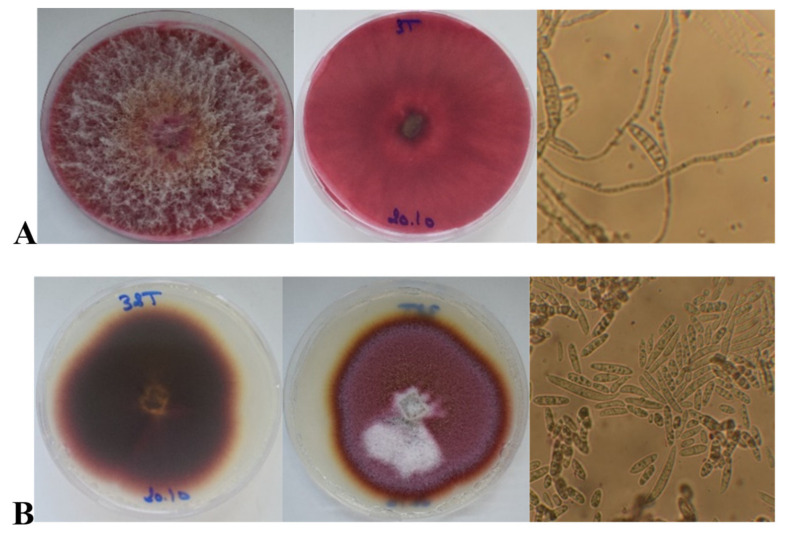
Morphological characteristics of *Fusarium* sp. isolated from asymptomatic Tunisian durum wheat grains. Observation of mycelial colonies and macroconidia grown on PDA. (**A**) 3T isolate representing the first morphotype after incubation at 25 °C for 7 days. (**B**) 32T isolate representing the second morphotype, after incubation at 25 °C for 11 days. Spores were observed under an optical stereomicroscope (Magnification 2000×).

In agreement with the encountered TCTB contamination, *Fusarium* sp. were isolated from grains of the durum wheat variety Khiar harvested in 2018 but not from the other samples. Most of the samples were contaminated with *Alternaria* sp. Seven *Fusarium* sp. colonies were obtained and purified using monosporal cultures. On PDA, two types of *Fusarium* mycelial colonies were observed (Figure 1).

Five isolates shared the first morphotype characterized by a pigmented mycelium that invaded the total surface of the 90 mm Petri dishes after 5 d of incubation at 25 °C and an aerial light mycelium with the presence of a brown/orange colored region in the center (sporodochia). The microscopic observation showed the presence of macroconidia widest at the midpoint of the macroconidium. The apical cell was rounded and blunt and the basal cell was notched and without a distinct foot shape. Four to five septa were observed. No microconidia were observed.

**Figure 2:**
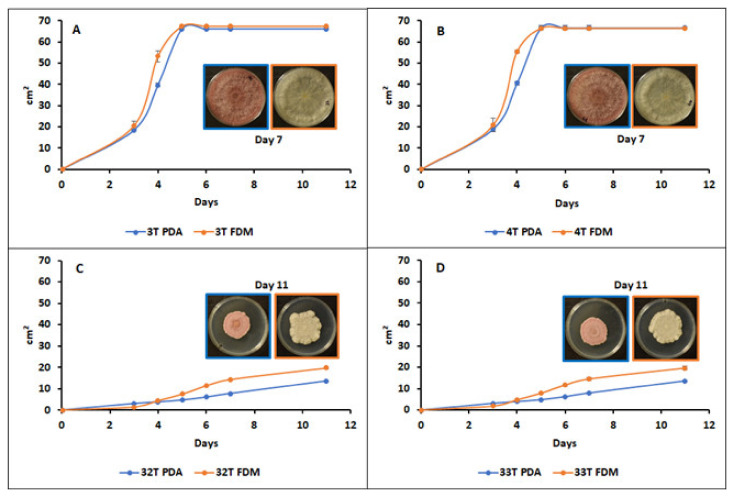
Growth kinetics and mycelial colony aspects of four isolated *Fusarium* strains ((**A**) = 3T, (**B**) = 4T, (**C**) = 32T, and (**D**) = 33T) on PDA and FDM-agar media. Values are means of 3 replicates. Error bars are ± standard deviation.

The two other isolates showed a compact flat red pigmented mycelium with no fluffy aerial parts. The observation was conducted for 11 days and the mycelium colonies did not invade the total surface of the Petri dishes, with the appearance of a red to brownish pigment in the agar. The spore observation showed an abundant production of oval microconidia with one to two septa. Some observed macroconidia presented three to four septa. It was also observed that when cultivated on FDM-agar, both morphotypes did not produce red pigments as on PDA, but their differences in mycelial aspects and colonization abilities were obvious (Figure 2).

The mycelial growth rates on both culture media helped to discriminate between the two sets of strains. It can be observed that 3T and 4T covered the plates after 5 days of incubation at 25 °C, both on FDM-agar and PDA (Figure 2A,B). The two other isolates, 32T and 33T, showed slower growth. Furthermore, almost a 30% higher growth was observed on FDM-agar than on PDA after 11 days, the colonies reached 19.7 cm² on FDM-agar and 13.5 cm² on PDA (Figure 2C,D).

Based on cultural and morphological characteristics of the colonies and conidia, and the growth rates, the group of isolates composed by 3T and 4T were identified morphologically as *F. graminearum* or *F. culmorum*. Molecular analyses based on ITS and *β-tub* gene sequences confirmed the identification of the isolates. They are members of the *F. sambucinum* species complex (152 hits each for the *β-tub* partial sequence) and were identified either as *F. culmorum* with the *β-tub* gene sequences, or as *F. graminearum* with ITS sequences that were identical (Table S1). Concerning the isolates 32T and 33T, using both their phenotypes and molecular analyses, they were identified as *F. acuminatum*, belonging to the *F. tricinctum* species complex with both ITS and *β-tub* gene sequences (Table S1). Their ITS sequences were identical, whereas their *β-tub* gene sequences varied.

**Figure 3:**
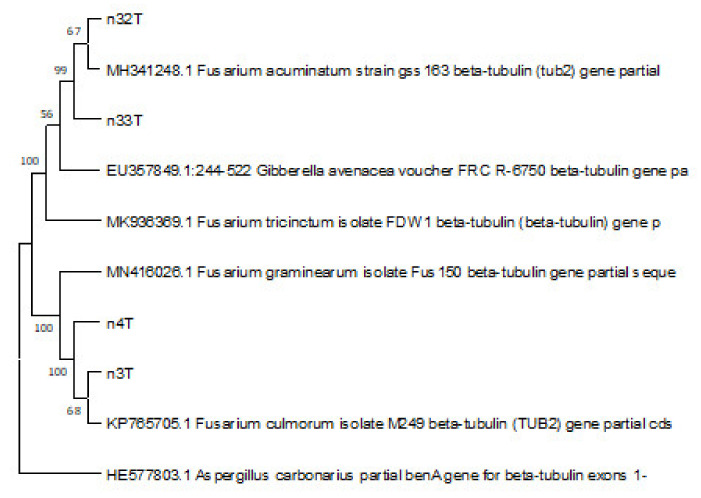
Dendrogram of similarity and difference of *β-tub* gene fragment sequences of the studied *Fusarium* species (species name and sample number are given) constructed using the UPGMA method. Stability was determined by bootstrap analysis (100 alternative dendrograms), the percentage of replicate trees in which the associated taxa clustered together in the bootstrap test (100 replicates) are shown next to the branches.

**Figure 4:**
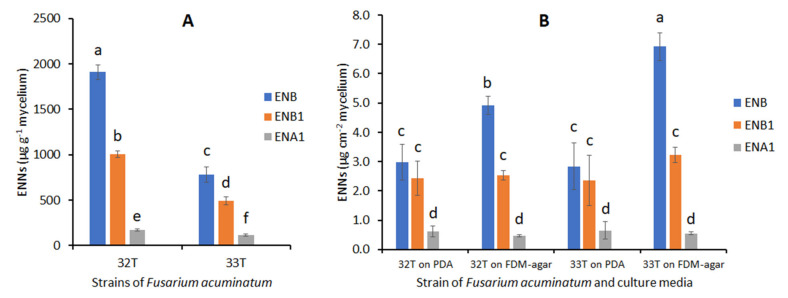
Accumulation of enniatins by *F. acuminatum* 32T and 33T in different culture media measured after 11 days of culture at 25 °C. (**A**) = culture in FDM liquid medium, (**B**) = culture on PDA and FDM-agar medium. Values are means of triplicates. Error bars are ± standard deviation. Means with the same lowercase letter are not significantly different (*p* < 0.05).

The multiple sequence alignment and the data in a phylogenetic tree for *β-tub* gene partial sequences of the four studied strains and referenced sequences (Figure 3) showed two distinct clusters with high support of the bootstrap test. It confirmed that 3T and 4T clustered with *F. culmorum* and to a lesser extent with *F. graminearum*. Combining data, the isolates 3T and 4T were determined as *F. culmorum*. Figure 4 also confirmed the clustering of 32T and 33T with members of the *F. tricinctum* species complex and their identity as *F. acuminatum*. The GenBank accession numbers of the sequences used are *F. culmorum* 3T—ON128289, *F. culmorum* 4T—ON128290, *F. acuminatum* 32T—ON128291, *F. acuminatum* 33T—ON128292. To the best of our knowledge, *F. acuminatum* has rarely been reported on cereals in Tunisia.

### 3.3. Production of Enniatins by the Isolates of F. acuminatum

The ENNs’ analysis was performed on the liquid FDM, FDM-agar, and PDA at the end of the incubations for 11 days at 25 °C. Both *F. acuminatum* 32T and 33T were able to produce ENB, ENB1, and ENA1 (Figure 4). No ENA was produced. ENB was the major ENN produced in liquid FDM (1910 and 782.5 µg g^−1^ of biomass for 32T and 33T, respectively) followed by ENB1 (1007 and 491.5 µg g^−1^ of biomass for 32T and 33T, respectively) and lower ENA1 levels were recorded (172.5 and 116 µg g^−1^ of biomass for 32T and 33T, respectively). The ENB levels of 61% and 56% of total ENNs were recorded for 32T and 33T, respectively. While, ENA1 recorded 6% and 9% of total ENNs for *F. acuminatum* 32T and 33T respectively. The total production of ENNs in cultures of 32T, 3089 µg g^−1^ mycelial biomass, was 2.2-fold that in cultures of 33T, 1390 µg g^−1^. For each ENN, the concentrations in *F. acuminatum* 32T cultures in liquid FDM were significantly higher (*p* < 0.05) than in 33T cultures.

In FDM-agar medium and PDA, ENA was always produced at the lowest concentrations, but the differences between ENB and ENB1 were not significant in PDA. In FDM-agar the ENB levels counted for 62% and 65% of total ENNs for 32T and 33T, respectively. *Fusarium acuminatum* 33T produced a significantly higher amount of total ENNs in the FDM-agar than in the PDA medium (10.7 µg cm^−^² and 6.0 µg·cm^−^² respectively). Due to its higher production of ENB1, the total ENNs’ production of *F. acuminatum* 33T in FDM-agar (10.7 µg cm^−^²) was significantly higher than the total ENNs’ production of 32T in the same medium (7.9 µg cm^−^²). Consequently, *F. acuminatum* 33T was chosen for assessing the effect of EOs on the production of ENNs.

### 3.4. Susceptibility of F. acuminatum to Essential Oils

**Figure 5:**
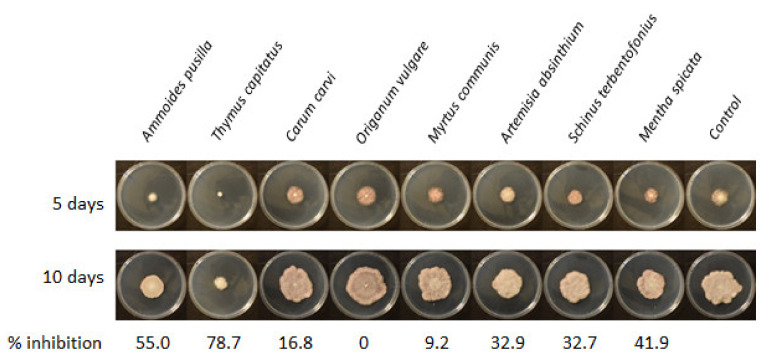
Susceptibility of *F. acuminatum* 33T to the antifungal activity of a set of essential oils from eight Tunisian plants diluted at 0.1 µL mL^−1^ in FDM-agar, incubated at 25 °C for 10 days.

The susceptibility of the isolate *F. acuminatum* 33T to different EOs was assessed in the FDM-agar medium. It was observed above that the strain produced a dense mycelial colony with a relatively low growth rate. This mycelial growth was susceptible to the antifungal activity of EOs, but at different levels, depending on the EO (Figure 5). It was recorded that two out of eight EOs diluted at 0.1 µL mL^−1^ in FDM-agar induced more than 50% inhibition of mycelial growth at 10 days, those being *Ammoides pusilla* EO (AP) and *Thymus capitatus* EO (TC) with 55% and 79% inhibition, respectively. The other EOs tested in this experiment showed no or lower effects (<10% inhibition) (Figure 5). Using AP EO concentrations from 0.05 to 0.15 µL mL^−1^ in FDM-agar medium, the inhibition of mycelial growth of *F. acuminatum* 33T was 18.9% and 94.8% at 0.05 to 0.15 µL mL^−1^, respectively, and the IC50 was evaluated at 0.06 µL mL^−1^. The effect of TC EO on 33T was studied previously and the IC50 was evaluated to be 0.01 µL mL^−1^ [14].

**Figure 6:**
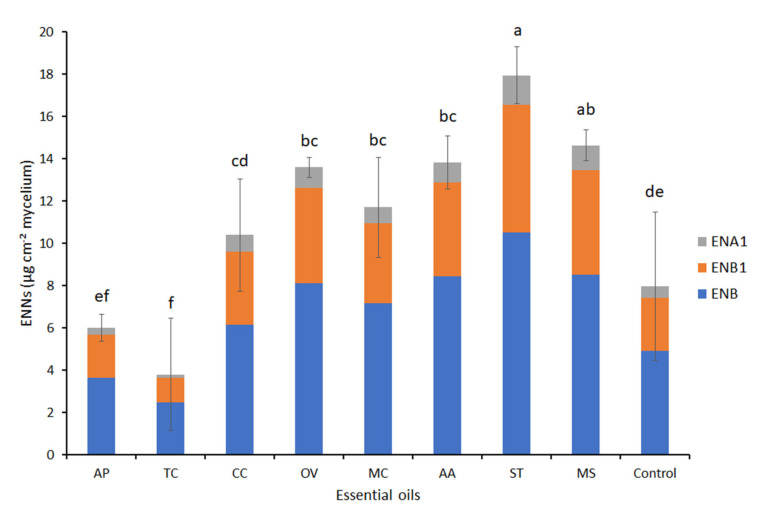
Effect of essential oils (EO) extracted from eight Tunisian plants, diluted at 0.1 µL mL^−1^ FDM-agar, on the production of enniatins by *F. acuminatum* 33T. Values are means of triplicates. Error bars are ± standard deviation. Means with the same lowercase letters are not significantly different (*p* < 0.05). ENB1 = Enniatin B1, ENB = Enniatin B, ENA1= Enniatin A1, ENA = Enniatin A. AP = *Ammoides pusilla*, TC = *Thymus capitatus*, CC = *Carum carvi*, OV = *Origanum vulgare*, MC = *Myrtus communis*, AA = *Artemisia absintum*, ST = *Schinus terbentofonius*, MS = *Mentha spicata*.

As in Figure 4B, the isolate 33T did not produce ENA in the FDM-agar medium (control) and 62% total ENNS were ENB; less than 10% were ENA1 (Figure 6). The ratios between the ENNS were not significantly affected by the EO treatments but the produced levels varied. Significant induction of the production of ENNs was observed with EOs having no or low effects on mycelial growth rates (inhibition < 40%), except *C. carvi* EO, which did not differ from the control (Figure 6), while a significant decrease in ENNs’ accumulation was observed when *F. acuminatum* 33T was exposed to TC EO treatments with a reduction level reaching 52%. With AP EO, the reduction level was 26%, but the difference with the control was not significant (Figure 6).

## 4. Discussion

The dry climatic conditions around the anthesis period of the years 2017 and 2018 in Northern Tunisia (Béjà) were not conducive to the development of FHB, and no symptoms were recorded on the experimental parcels used for characterizing the 13 wheat cultivars sampled in this study. In our attempt to isolate *Fusarium* sp. from these asymptomatic harvested grains, only one sample was positive, and isolates of *F. culmorum* and *F. acuminatum* were obtained. This sample was also the only one containing DON contamination. The presence of TCTB in asymptomatic grains is currently observed. In a Spatio-temporal model of the multifaceted virulence strategy deployed by the advancing *F. culmorum*, it was proposed that asymptomatic infection occurs with high induction of the TRI genes involved in DON biosynthesis and accumulation [21]. The obtained strain of *F. culmorum* was probably responsible for the DON accumulation in the grains studied in the present work.

The colonization of spikes by at least two FHB pathogens producing the same or different mycotoxins is a common observation. Audenaert et al. [22] reported a clear association between *F. poae* producing NIV and *F. avenaceum* producing ENNs in symptomatic ears collected in Flanders in 2007. However, the co-occurrence of *F. acuminatum* with *F. culmorum* in wheat grains is not a common observation since *F. acuminatum* is infrequently found in wheat grains. *Fusarium* species have been divided into 23 informal multispecies lineages, termed species complexes [23]. *Fusarium acuminatum* is a member of the *F. tricinctum* species complex along with *F. avenaceum* and *Fusarium tolurosum* as well as other species. The members of the *F. tricinctum* species complex have a wide geographic distribution and can occur on extremely diverse plants, often without causing severe disease [5]. They are classified as FHB contributors but also as causing agents of *Fusarium* foot rot. *Fusarium acuminatum* is known to determine significant reductions in the germination ratio and vegetative vigor of durum wheat seedlings [24] and as one of the causing agents of dryland root rot of winter wheat in Colorado [25]. It exhibited weak aggressiveness on wheat seedlings and had a mean crown rot activity in China where significant correlations of its occurrence in diseased wheat stems with global climatic variables were observed [26]. *Fusarium acuminatum* isolates were weakly pathogenic in an FHB pathogenicity assessment on different durum wheat varieties in Tunisia [6].

The obtained isolates were identified by both morphological and molecular tools (Figure 1, Figure 2 and Figure 3). Overall, the *β-tub* gene site was more effective in distinguishing and identifying the species under study than the ITS. The morphologic identification of two isolates of *F. acuminatum* conducted in this work was in line with the findings of Logrieco et al. [27] who identified a slow-growing type of *F. acuminatum* with carmine-red pigmentation and the production of heterogeneous macroconidia (mostly three to four septa). Lesli and Summerell [17] also described *F. acuminatum* as a relatively slow-growing species and mentioned the formation of red pigments, sometimes brown, in the agar and that the presence of macroconidia with three and four septa was not uncommon [18]. The pigment formation was confirmed by Burgess and Forbes [28]. The abundant production of microconidia was also described in the work of Rabie et al. [29].

*Fusarium acuminatum* has been recovered from cereals in Canada, North Carolina, and Mediterranean countries, but at a lower frequency compared to *F. avenaceum* and *F. tricinctum* [30,31,32]. In Italy, *F. acuminatum* was reported to be among the most harmful *Fusarium* species for durum wheat production causing *Fusarium* foot rot. It was detected within 50% of the analyzed soils in Sicily and seemed not to be affected by the suppressive effects of biofumigation with brassicaceous green manures [33]. Studying *Fusarium* species colonizing the lower stems (crowns) of bread and durum wheat from different regions of Turkey, Gebremariam et al. [34] isolated *F. acuminatum* with a frequency of 14%, mostly related to crown rot. The members of the *F. tricinctum* species complex are also contributors to FHB. *Fusarium acuminatum* has been isolated from Italian durum wheat grains from three cultivation areas [31], and from symptomatic wheat and barley grains [32]. In this latter study, the authors identified *F. acuminatum* isolates from Italy (growing season 2017–2018), using molecular tools. In a previous study, when collecting wheat grains on FHB symptomatic spikes at the filling stage from 11 fields in Northern Tunisia during the 2004 and 2007 crop seasons, *F. acuminatum* was found in four different fields with always low percentages compared to the most common *Fusarium* species, these being *F. culmorum*, *F. avenaceaum*, and *F. equiseti* [6]. *Fusarium acuminatum* was not detected in other studies dealing with Tunisian wheat grains [6,7,8]. It was rarely found in other Tunisian grains such as sorghum in a study where 1 out of 53 *Fusarium* sp. isolates identified was related to *F. acuminatum* [35]. Due to the scarcity of information about *F. acuminatum* in wheat grains in Tunisia, its potential as a harmful *Fusarium* species was highlighted by observations in the Mediterranean conditions in Sicily [33], and its capacity to produce emerging mycotoxins (Beauvericin, ENNs, Fusaproliferin, and Moniformin) [5], it was of concern to characterize the production of at least one of these mycotoxins by the isolates we obtained from a Tunisian durum wheat sample.

In our study, ENA and ENB1 co-occurred with DON in the wheat sample from which the strains of *F. acuminatum* were isolated. However, ENNs were detected in all the wheat cultivars in the two growing seasons (Table 1), and the highest ENNs levels were found in the sample of the Khiar variety contaminated with DON, these being 524 and 41 mg Kg^−1^ for ENA and ENB1, respectively. When investigating the presence of ENNs, BEA, and FUS in cereals and derived products from Tunisia, Oueslati et al. [10] found also that all the analyzed wheat samples were contaminated with ENNs, at a maximum concentration of 532 mg Kg^−1^. Using the same extraction procedure and LC-DAD analysis on breakfast and infant cereals from Morocco, Mahnine et al. [36] found a maximum total ENNs value of 1264 mg Kg^−1^. Studies on ENNs’ contamination in wheat grains and wheat products published recently were most of the time performed using quantification by LC-MS/MS in multi-mycotoxin analysis and reported quite lower levels than in works using LC-DAD analysis. Investigating 470 bread wheat and 260 durum wheat samples from the French harvests of 2012, 2013, and 2014, and using a quantification by LC-MS/MS, Orlando et al. [37] found an occurrence of ENNs ranging from 53% (2014) to 91% (2012), and mean values ranged from 47 to 142 µg Kg^−1^ for bread wheat and from 55 to 596 µg Kg^−1^ for durum wheat. In a long-term field survey using HPLC-QqQ-MS/MS in The Netherlands, ENA1, ENB, and ENB1 were detected in 14.2%, 46.6%, and 26.2% of the analyzed samples, respectively [38]. In Algeria, 21 out of 30 (70%) wheat samples were contaminated with ENB1 at concentrations from 19 to 4569 µg Kg^−1^, and seven samples were contaminated with ENA at concentrations ranging from 8 to 88 µg Kg^−1^ [39]. ENA1 and ENB were also detected in many samples. In the present study on asymptomatic grains, no ENNs were detected using quantification by LC-MS/MS in samples showing concentrations of ENA ranging from 320 to 930 mg Kg^−1^ when the LC-DAD method was used. This agrees with the absence of *Fusarium* sp. isolation. Metabolites extracted from grains may have interfered with the quantification of ENNs, and data have to be used with caution.

Despite the uncertainty of the accumulation of ENNs in the grains, the studied isolates identified as *F. acuminatum* proved to be able to produce three ENNs in vitro, ENA1, ENB, and ENB1 but not ENA (Figure 4). The difference in distribution between the four ENNS in naturally contaminated grains and in liquid or agar culture media may be due to differences in the regulation of the synthesis in various environments. In different works, *F. acuminatum* has been reported to be capable of producing ENA, A1, B, B1, B2, B3, B4, P1, and P2 [40]. Our results on ENNs’ production agree with those of Senatore et al. [32] who studied five strains of *F. acuminatum* from a culture collection and four new ones isolated in 2017–2018 from symptomatic wheat and barley grains in Italy. When incubated in vitro on rice for 10–12 days in the dark at 25 °C, ENB and ENB1 were detected at similar concentrations, and ENA1 at lower concentrations (1/3 that of ENB or ENB1 for six strains), but no ENA was detected. The results we obtained with two Tunisian strains of *F. acuminatum*, 32T and 33T, agree with this distribution of ENNs, both in liquid and agar media. In a previous work where *F. acuminatum* 33T was cultivated on FDM-agar medium, its level of ENNs’ production was 10-fold higher than for a strain of *F. avenaceum* cultivated under the same conditions [14]. Comparing the single *F. acuminatum* strain with 30 *F. avenaceum* they isolated in a survey of barley and wheat fields in Paraná state, Brazil, Peirera et al. [41] found *F. acuminatum* to be the strongest producer of ENNs. Despite its low occurrence, *F acuminatum* has to be considered as a significant source of mycotoxin contamination of crops, due to its high potential for ENNs’ production.

The antifungal activity of the same eight EOs from Tunisian plants used (Figure 5) here was previously tested against a strain of *F. avenaceum*, which was significantly more affected by AP and TC EOs than by the other EOs [15], as *F. acuminatum* 33T was in the present study (Figure 5). The mycelial growth inhibiting effect of TC EO was higher than that of AP EO for both species. The same level of inhibition of ENNs’ accumulation in the FDM-agar medium by *F. acuminatum* 33T was observed here for both EOs, whereas AP EO was more effective than TC EO for *F. avenaceum* [15]. The effectiveness of AP and TC EOs on two members of the *F. tricinctum* complex is a strength for their putative use for controlling these ENN producers and limiting the accumulation of the mycotoxins in plant products. The EOs represent promising biosourced products for the development of natural and eco-friendly alternatives to synthetic fungicides that are currently used. However, practically, the application of crude EOs in the field or at grain storage faces several limits. The production of EOs is limited by the extraction yield from aromatic plants and scaling-up would involve the production of large amount of plant material. New extraction methods with higher yields are now available. Subcritical water extraction (SWE) avoids the loss and degradation of volatile and thermolabile compounds, is low cost, and has favorable environmental impacts [42]. Another limit is that essential oils are very volatile substances and are sensitive to the exposure to oxygen in the air and to UV light [43]. The development of a system that protects the EOs from the oxidation and increases the evaporation time is necessary to potentialize them as biofungicides. Encapsulation of the essential oils in different matrixes has been studied to reach this aim. This method can improve their antifungal activity and increase the release period [44,45]. Thus, nanoencapsulation enables the use of lower doses of EOs to obtain the same results as the EOs on a free state. The latter point is primordial for the development of different formulations to potentialize the EOs as the scale-up of their production in the aim of field application is a complicated task. Finally, the determination of the contribution of different compounds constituting an EO in its biological activity provides a better understanding of the different interactions among the compounds and their activity [15,44]. Hence, formulations focusing on the mixture of the main active compounds in their synthetic form is a promising area of investigation to provide biofungicide alternatives.

It is worth mentioning that, as with other cyclodepsipeptide analogs, ENNs are involved in plant–pathogen interactions, and they are detected in a range of foods or feeds originating from many countries. As they belong to the mycotoxin group, they may be dangerous for human health, but they also possess antimicrobial, insecticidal, antifungal, and antibiotic activities, which may help to develop new drugs, and due to their cytotoxicity, they may be applied in anticancer future therapies [40]. *Fusarium acuminatum* 32T and 33T are good candidates for producing ENNs in large quantities for medical applications.

## 5. Conclusions

*Fusarium acuminatum,* a member of the *F. tricintum* species complex was shown to be a co-contaminant, with *F. culmorum*, of asymptomatic wheat grains in Tunisia. This may contribute to the co-accumulation of ENNs and TCTB in cereal crops. The isolates we obtained were able to produce significant quantities of ENB and ENB1 in vitro. This *Fusarium* species should be carefully considered in future works on grain mycotoxin contamination in Tunisia and other Mediterranean countries and the development of strategies for its control is a challenge. In this study, two strains of *F. acuminatum* were susceptible to the antifungal activities of essential oils extracted from different Tunisian plants, but those limiting less than 50% of the mycelial growth under the condition of the experiment tended to stimulate the production of ENNs and are not recommended for the control of *F. acuminatum*. However, the essential oils extracted from *T. capitatus* significantly inhibited both the mycelial growth and the production of ENNs. It could be used for developing a biocontrol product against *F. acuminatum*.

## Figures and Tables

**Table 1:** List of primers used in the work.

Reference Gene		Primer Sequences (5′-3′)	References
***β-tubulin***	β-tubulin 261F	GGTAACCAAATCGGTGCTGCTTTC	[19]
	β-tubulin 536R	GATTGACCGAAAACGAAGTTG	
**ITS**	ITS1 F	TCCGTAGGTGAACCTGCGG-	[20]
	ITS4	TCCTCCGCTTATTGATATGC	

**Table 2:** Levels of enniatin analogues (mg Kg^−1^) in Tunisian wheat grains from different varieties comparing crop years (2017 vs. 2018) and cultures (Durum vs. Bread varieties).

	Years			
	2017	2018	2017	2018
Number of varieties	13	10	13	10
	Enniatin A		Enniatin B1	
Mean	549 ± 147	551 ± 193	33 ± 6.1	43 ± 16.3
Maximum	716	930	47	77
Minimum	ND	ND	26	26
	**Wheat Varieties**			
	**Durum Wheat**	**Bread Wheat**	**Durum Wheat**	**Bread Wheat**
Number of samples	15	8	15	8
	Enniatin A		Enniatin B1	
Mean	618 ± 209	505 ± 97.4	35 ± 5.8	43 ± 17.8
Maximum	931	618	43	77
Minimum	259	340	25.1	18

± standard deviation, ND: Not detected. Enniatins were analysed by HPLC/DAD with LOD = 0.01 mg Kg^−1^ of grain and LOQ = 0.5 mg Kg^−1^ of grain.

## Data Availability

Not applicable.

## Supplementary Materials

The following supporting information can be downloaded at: https://www.mdpi.com/article/10.3390/jof8050458/s1, Table S1: Molecular identification of the *Fusarium* sp. isolates based on ITS and β-tubulin sequence analysis.
