# Supplementary material for: Characterization of Fusarium acuminatum: A Potential Enniatins Producer in Tunisian Wheat
Source: J Fungi (Basel). 2022 Apr 28;8(5):458. doi: 10.3390/jof8050458 (PMC9144410; doi:10.3390/jof8050458)
Supplement: Supplementary file 1 [file jof-08-00458-s001.zip › jof-1691861-supplementary.pdf]

Table S1. Molecular identification of the Fusarium sp. isolates based on ITS and β -tubulin sequence analysis.

Isolate	β-tub sequence			
	Lineage organism	Nb of hits	Lineage organism *	Nb of hits
**3T**	*F. sambucinum* species complex	152	*F. pseudograminearum*	78
**4T**	*F. sambucinum* species complex	152	*F. pseudograminearum*	78
**32T**	*F. tricinctum* species complex	66	*F. acuminatum*	55
**33T**	*F. tricinctum* species complex	64	*F. acuminatum*	51
	Best nr database similarity hit			
	Taxon	E value	ID %	GenBank Accession No.
**3T**	*F. culmorum*	4e-100	100	LT598662.1
**4T**	*F. culmorum*	5e-99	99.04	LT598662.1
**32T**	*F. acuminatum*	5e-68	96.88	MH341248.1
**33T**	*F. acuminatum*	8e-92	98.48	MH341248.1
	**ITS sequence**			
	Lineage organism	Nb of hits	Lineage organism *	Nb of hits
**3T**	Sordariomycetes	101	*F. graminearum*	49
**4T**	Sordariomycetes	101	*F. graminearum*	56
**32T**	*F. tricinctum* species complex	72	*F. acuminatum*	54
**33T**	*F. tricinctum* species complex	95	*F. acuminatum*	49
	Best nr database similarity hit			
	Taxon	E value	ID %	GenBank Accession No.
**3T**	*F. graminearum*	0	99.76	MT598163.1
**4T**	*F. graminearum*	0	99.76	MT598163.1
**32T**	*F. acuminatum*	0	100	MT635295.1
**33T**	*F. acuminatum*	0	100	MT635295.1

* Species with the highest number of hits
